# Biomechanical strain patterns in Meckel’s cartilage underlie mandibular variation in FGFR2 mouse models

**DOI:** 10.1002/ar.70371

**Published:** 2026-10-06

**Authors:** Fred R. Foster, Alexandre Freire, Ana Cláudia Rossi, Jordan Wilson, Joan T. Richtsmeier, Kazuhiko Kawasaki, Felippe B. Prado, Susan M. Motch Perrine

**Affiliations:** 1 Department of Anthropology, The Pennsylvania State University, University Park, Pennsylvania, USA; 2 Department of Biosciences, Piracicaba Dental School, University of Campinas (UNICAMP), Piracicaba, Brazil; 3 Department of Otolaryngology – Head and Neck Surgery, Penn State College of Medicine, Hershey, Pennsylvania, USA; 4 Department of Medicine, Penn State College of Medicine, Hershey, Pennsylvania, USA

**Keywords:** biomechanical modeling, craniosynostosis syndromes, FGFR2 sequence variants, mandibular morphology, Meckel’s cartilage development

## Abstract

Sequence variations in fibroblast growth factor receptor 2 (FGFR2) are associated with craniosynostosis syndromes, disrupting craniofacial development, yet early mandibular alterations remain poorly characterized. This study examined Meckel’s cartilage (MC) and mandibular morphology in mouse models of Apert (*Fgfr2*^+/*P253R*^) and Crouzon (*Fgfr2c*^*C342Y*/+^) syndromes using Euclidean distance matrix analysis and finite element modeling at embryonic days 14.5 and 16.5. Apert mice exhibited significant MC shortening and elevated, widespread strain in the growth center area correlating with mandibular shortening and asymmetry. In contrast, Crouzon mice showed minor MC changes and localized strain peaks with larger mandibles. These biomechanical perturbations in Meckel’s cartilage are associated with distinct mandibular phenotypes, consistent with a bidirectional relationship in which altered strain environments may both influence and reflect mandibular dysmorphogenesis during early development. The findings enhance our understanding of the biomechanical basis of mandibular anomalies associated with FGFR2 mutations, informing future investigations into craniofacial morphogenesis.

## 1 | INTRODUCTION

Apert and Crouzon syndromes are classified as fibroblast growth factor receptor (FGFR)-related craniosynostosis syndromes, with all affected individuals carrying a pathogenic variant of FGFR2. These conditions are characterized by premature fusion of one or more craniofacial sutures, contributing to abnormal development of the cranial vault, cranial base, and facial skeleton (Wenger et al., 1998, Oct 20 [Updated 2020 Apr 30]). Under-development of the maxilla, zygomatic arches, and other facial structures leads to midfacial hypoplasia, which often contributes to functional impairments including malocclusion (misalignment of the teeth) and upper airway obstruction (Zeppieri et al., 2025). Although mandibular dysmorphology and dental crowding are present in Apert and Crouzon syndromes, these features are generally less pronounced than those affecting the maxilla and upper jaw (Krarup et al., 2005). In Crouzon syndrome, the mandible tends to become retrognathic (positioned posteriorly relative to the cranial base) earlier than changes in mandibular shape, whereas in Apert syndrome, mandibular shape deformity occurs simultaneously with alterations in the spatial relationship between the mandible and the cranial base (Lu et al., 2019).

Meckel’s cartilage (MC) serves as a cartilaginous precursor and structural support for later mandibular bone formation (Pitirri et al., 2022). MC develops around embryonic day (E)12 when neural crest-derived cells condense and differentiate down a chondrogenic pathway (Chai et al., 2000). By E13, chondrocytes in MC are organized into two rods that merge at the anterior midline of the lower jaw to form a V-shaped structure at E14, prior to the onset of mandibular ossification (Ramaesh & Bard, 2003). At E14.5, a population of cells lateral to the rods of MC initiates bone formation, which then extends anteriorly and posteriorly to form the mandibular corpus (Abe et al., 2016; Ramaesh & Bard, 2003). As ossification progresses, the anterior portion of MC undergoes hypertrophy and mineralization, contributing to the size and shape of the mandible (Svandova et al., 2020). Given the close developmental relationship between MC and the mandible, it is plausible that deviations in MC growth contribute to the mandibular shape deformities observed in FGFR2- related craniosynostosis syndromes, including Apert and Crouzon syndromes (Lu et al., 2019; Reitsma et al., 2012).

Previous research has explored changes in the growth of MC and the mandible during embryogenesis using mouse models for FGFR-related syndromes. Size and shape changes have been observed in MC and the mandible when the *Fgfr2c*^*C342Y*/+^ mouse model for Crouzon syndrome was compared with its genetically unmodified (unaffected) *Fgfr2c*^+/+^ littermate control (Pitirri et al., 2022). Notably, differences in MC were detected before the earliest appearance of bone at E13.5 and persisted through the period when MC and the mandible are in direct contact, up to E17.5. Mandibular differences in affected mice were consistent with those observed in humans (Lu et al., 2019; Reitsma et al., 2012). The presence of MC differences prior to mandibular bone formation in affected versus unaffected individuals suggests that alterations in MC size and shape may drive changes in mandibular prognathism in FGFR-related syndromes.

Changes in the size, shape, and anterior extension (prognathism) of MC are likely to influence mandibular development through biomechanical interactions, especially once the mandible contacts the perichondrium of MC during embryogenesis (Brunt et al., 2015; Farahat et al., 2021; Farahat et al., 2022). To better understand these genetic and morphological changes, we investigated the biomechanical interactions of stress and strain experienced by MC as it changes shape during development (Brunt et al., 2015). In this study, we examined the distribution of stress and strain in MC using mouse models for Apert and Crouzon syndromes, focusing on embryonic days E14.5 and E16.5 that span the onset of mandibular bone formation. We hypothesize that the magnitude and distribution of stress and strain in MC will differ significantly between affected and unaffected mice, and that these differences may help explain the mandibular dysmorphologies associated with Apert and Crouzon syndromes.

## 2 | MATERIALS AND METHODS

### 2.1 | Mouse models

We tested our hypothesis using *Fgfr2*^+/*P253R*^ Apert syndrome mice (Wang et al., 2010) and *Fgfr2c*^*C342Y*/+^ Crouzon syndrome mice (Eswarakumar et al., 2004). These mice carry a heterozygous missense variant in *Fgfr2*, and the variant is predicted to substitute an amino acid residue of the FGFR2 protein, causative for Apert syndrome (P253R) and Crouzon syndrome (C342Y) phenotypes, respectively. All analyses using mice were approved by the Pennsylvania State University Animal Care and Use Committee (IACUC # PRAMS200446558) and complied with animal welfare guidelines. A total of 46 specimens were collected at E14.5 and E16.5 based on timed matings and after euthanizing pregnant dams using inhalation anesthetics. The distribution of the sample by genotype and embryological age is shown in Table 1. The developmental age of each E14.5 specimen was confirmed using the eMOSS system (Musy et al., 2018). All specimens were stored in 90% methanol solution until further preparation and scanning. PCR amplification was used to determine the genotype in the mouse models carrying the mutation associated with Apert syndrome (*Fgfr2*^+/*P253R*^) and their genetically unmodified (unaffected) *Fgfr2*^+/+^ littermate controls and those carrying the mutation associated with Crouzon syndrome (*Fgfr2c*^*C342Y*/+^) and their unaffected *Fgfr2c*^+/+^ littermate controls (Eswarakumar et al., 2004; Wang et al., 2010). Unaffected littermates refers specifically to littermates lacking the disease-associated *Fgfr2* mutation under investigation; affected littermates refers specifically to littermates carrying the disease-associated *Fgfr2* mutation. This designation is relative to the modeled disorder and does not imply that these animals lack all genetic modifications or are unaffected in every biological respect.

### 2.2 | Imaging and data collection

All micro-CT images were collected at the Center for Quantitative Imaging at the Pennsylvania State University using a General Electric v|tome|x L300 Micro-Nano CT scanner. Two sets of scans were produced for each specimen: one scan targeting bone and one scan phosphotungstic acid-enhanced (PTA-e) micro-computed tomography (micro-CT) to target soft tissues. Following previously published lab protocols (Lesciotto et al., 2020), all specimens were stored in 7% PTA solution for 8–15 days prior to scanning and were mounted in a 1:1 mixture of polyester and paraffin wax prior to PTA-e scanning. The scan parameters for each set of scans are given in Table 2. Images were reconstructed using Datos|x 2 Reconstruction (version 2.14.0; RTM, Waygate Technoloies, Wunstorf, Germany), a proprietary software designed to manage the entire computed tomography process chain including data acquisition, volume reconstruction, and evaluation, as a 32-bit volume on 2024 × 2024 grid and resampled to a 16-bit volume in FiJi (version 2.15) prior to analysis. During bone scanning procedures, a hydroxyapatite bone phantom was scanned alongside each group of specimens, and an approximate bone mineral density threshold of 85 mg/cm^3^ was calculated. Image data were passed through a median filter, and the bone mineral density threshold was used to segment bone and visualize an isosurface of the mandible for each specimen in Avizo 2021.1 (ThermoFisher Scientific, Waltham, MA). Cartilage was segmented from processed PTAe micro-CT scans using an automatic deep learning-based approach (Zheng et al., 2020). This method was previously used to effectively segment the more complicated structures in the chondrocranium of embryological mice in a recent study of the *Fgfr2c*^*C342Y*/+^ mouse model of Crouzon syndrome (Motch Perrine et al., 2022). We use the same method to segment cartilage here, which includes a two-part approach with (1) sparse hand annotation of ground truth data by anatomical experts and (2) limited corrections after automatic segmentation to fine-tune the model. This produced 3D models of the cartilaginous skeleton for each specimen that were used to create isosurfaces of MC in Avizo 2021.1. Representative samples of 3D isosurfaces for MC and mandible bone are shown in Figure 1.

### 2.3 | Statistical analysis

To determine differences in the shape of the mandible and MC between groups, which are defined in Table 1, we used Euclidean distance matrix analysis (EDMA), a morphometric method used to statistically compare the physical shape and size of biological objects or structures using 3D landmark data (Lele & Richtsmeier, 2001). This method offers a coordinate-free approach to shape comparisons by testing for differences in the distances associated with all 3D landmark pairs using a bootstrapped hypothesis test, and by generating nonparametric bootstrapped confidence intervals for the linear distance defined by each landmark pair (Lele & Richtsmeier, 1995; Lele & Richtsmeier, 2001). EDMA confidence intervals for form differences are built using bootstrap methods. We used 10,000 resamples of the bootstrap reference sample (numerator) in each of our analyses. Statistical significance is determined by checking if the interval for a pairwise landmark distance ratio contains the null value of 1.0 (indicating no difference). If the entire 95% confidence interval lies above or below 1.0, the difference is significant (*α* = 0.10) (Lele & Richtsmeier, 1995). Details of the approach and statistical tests have been published elsewhere (Lele & Richtsmeier, 1995; Lele & Richtsmeier, 2001).

To determine differences in relative growth between groups we use an extension of EDMA that estimates growth as a 3D comparison of form between two age groups within genotypes. 3D landmark locations are used to estimate MC or mandibular morphology for each age group and ratios of like linear distances are used to estimate the relative changes in geometry that occur due to growth (Richtsmeier & Lele, 1993). Growth estimates for each genotype are then compared statistically. In our application, an estimate of the relative growth from E14.5 to 16.5 is estimated for each genotype. Differences in relative growth between genotypes are defined by a linear distance-by-linear distance comparison and then statistically evaluated. EDMA confidence intervals for growth differences are built using bootstrap methods as described above.

The three-dimensional (3D) location of biological landmarks (Figure 2 and https://getahead.la.psu.edu/landmarks/) were recoded using the 3D isosurfaces of the mandible and MC for each specimen using 3D Slicer (4.10; https://www.slicer.org) (Fedorov et al., 2012). Landmarks of the mandible and MC are defined in Tables 3 and 4, respectively. Individual linear distances were determined to be significantly different between groups based on their associated nonparametric bootstrapped 90% confidence intervals (Lele & Richtsmeier, 1995). We tested for differences in the shape of MC in *Fgfr2*^+/*P253R*^ Apert syndrome mice and *Fgfr2c*^*C342Y*/+^ Crouzon syndrome mice as compared to their model appropriate unaffected controls at E14.5 and E16.5. We tested for differences in the shape of the mandible between affected and unaffected specimens at E16.5 for *Fgfr2*^+/*P253R*^ Apert syndrome mice and *Fgfr2c*^*C342Y*/+^ Crouzon syndrome mice.

Two sets of EDMA were performed on the mandibles; one using six landmarks on the left hemimandible to test for significant differences in the anteroposterior and superoinferior directions, and one using three bilaterally paired landmarks (six total) to test for significant differences in the mediolateral direction. To test for differences associated with growth, we used growth difference matrix analysis (GMDA) (Richtsmeier & Lele, 1993) to compare the shape of MC in E14.5 and E16.5 specimens, for both the Apert and Crouzon samples. GDMA is a modification of EDMA that compares a younger sample to an older sample within a genotype to estimate the growth pattern and then statistically compares the growth pattern of mice that carry the genetic variant to those that do not. All EDMA and GDMA analyses were performed using WinEDMA (University of Missouri-Kansas City, Kansas City, MO) (Cole, 2002) and EDMAinR (University of Alberta, Edmonton, Canada) (Solymos et al., 2023).

### 2.4 | Finite element analysis of shape change

Finite element analysis (FEA) was used to describe the biomechanical consequences of size and shape changes in MC from E14.5 to E16.5. Prior to FEA, we performed generalized Procrustes analysis (GPA) of the landmark data to identify the specimen that was closest to the geometric mean for each developmental age-genotype group. GPA was performed in 3D Slicer (4.10) using the GPA module in the SlicerMorph extension (https://slicermorph.github.io/) (Rolfe et al., 2021). The geometric mean isosurfaces were then aligned in the same coordinate space using landmark data with the Fiducial Registration module in 3D Slicer (4.10).

The FEA protocol was performed at Faculdade de Odontologia de Piracicaba-Universidade Estadual de Campinas. The isosurfaces were exported in stereo-lithographic format (STL) and imported into 3-Matic v10 software (Materialise, Belgium). Initially, the isosurfaces were optimized to improve polygon quality, and then they were converted into volumetric finite element meshes composed of three-dimensional tetrahedral elements with quality *q* = 0.74 ± 0.1 (in a range from 0 to 1). The finite element meshes were transferred to Ansys v17.2 software (Ansys, Inc., Canons-burg, PA) for FEA.

Three sets of FEA were performed on the meshed models of MC to determine the distribution of stress and displacement when the size and shape changes from E14.5 to E16.5. Each set used a different approach to induce the change from E14.5 to E16.5 in the meshed models associated with affected and unaffected genotypes. The first set applied force vectors to the meshed models at the nodes that corresponded with the coordinates of landmarks at E14.5, to change its size and shape until it matched the meshed model at E16.5 (landmark-driven change). The second set applied force vectors to meshed models at E14.5 that correspond to the regions within MC associated with chondrocyte hypertrophy (growth center-driven). Chondrocyte hypertrophy occurs in MC when cartilage cells located just posterior to the intersection of the left and right rods (anterior process) begin to swell in size, driving morphological change during development (Foster, et al., in review). To identify the regions of hypertrophic chondrocytes, one specimen at each developmental stage and genotype was sectioned in the coronal plane to create 7 μm thick sections. A subset of these sections were stained using picrosirius red and alcian blue to show bone and cartilage, respectively (Gruber et al., 2007). Regions where chondrocytes showed early signs of cell size expansion and hypertrophy were then identified, and the orientation and approximate anatomical position of each of these sections was determined by comparison with the micro-CT data described above. Each section was aligned with the micro-CT data associated with the geometric mean specimen at the same developmental stage to establish the location of the expanding hypertrophic chondrocytes within MC. These locations were then mapped onto the corresponding meshed models and used to define the nodes that displace during growth from E14.5 to E16.5. The third set of FEA combined the landmark- and growth center-driven approaches.

## 3 | RESULTS

### 3.1 | Morphology of Meckel’s cartilage

#### 3.1.1 | Morphological differences of Meckel’s cartilage in *Fgfr2*^+/*P253R*^ Apert syndrome mice and unaffected *Fgfr2*^+/+^ littermate controls

The results of EDMA comparing *Fgfr2*^+/*P253R*^ Apert syndrome mice with their unaffected *Fgfr2*^+/+^ littermate controls at E14.5 revealed 17 linear distances including measurements of the anterior process, the lengths of the right and left rods (measured from both the anterior-most and posterior-most points of the anterior process), the superior–inferior lengths of the left and right malleus, and the widths (measured from the superior-posterior-most points of the left and right malleus) reduced by 5–27.4% in *Fgfr2*^+/*P253R*^ mice, representing an overall smaller MC (Figure 3a). Two linear distances in the most posterior portion of MC were significantly larger in *Fgfr2*^+/*P253R*^ mice compared to unaffected *Fgfr2*^+/+^ littermate controls (Figure 3a).

At E16.5, fewer significant differences were observed between *Fgfr2*^+/*P253R*^ and *Fgfr2*^+/+^ mice, with 2 linear distances describing the lengths of the right and left rods being 5% smaller in affected *Fgfr2*^+/*P253R*^ specimens as compared to their unaffected *Fgfr2*^+/+^ littermate controls. Only two linear distances in the most posterior portion of MC and the linear distance describing the anterior process were significantly larger (11–13%) in *Fgfr2*^+/*P253R*^ mice relative to their unaffected *Fgfr2*^+/+^ littermate controls at E16.5 (Figure 3b).

#### 3.1.2 | Morphological differences of Meckel’s cartilage in *Fgfr2c*^*C342Y*/+^ Crouzon syndrome mice and unaffected *Fgfr2c*^+/+^ littermate controls

At E14.5, *Fgfr2c*^*C342Y*/+^ Crouzon syndrome mice exhibited fewer form differences relative to their *Fgfr2c*^+/+^ littermate controls. Only two linear distances representing the anterior process (17.7%) and the superior–inferior length of the left malleus (17.8%) were larger in affected mice compared to unaffected mice (Figure 3c). By E16.5, six linear distances describing distances between the posterior aspects of the left and right MC rods (~8.5%) and the most posterior portion of MC (5.8–15.8%) were larger in *Fgfr2c*^*C342Y*/+^ mice as compared to their *Fgfr2c*^+/+^ littermate controls (Figure 3d).

#### 3.1.3 | Growth differences of Meckel’s cartilage in craniosynostosis model mice from E14.5 to E16.5

No significant growth differences were observed in MC between affected and unaffected specimens of either the Apert or Crouzon syndrome model mice from E14.5 to E16.5.

### 3.2 | Morphology of the mandible

#### 3.2.1 | Morphological differences of *Fgfr2*^+/*P253R*^ Apert syndrome model affected specimens and unaffected *Fgfr2*^+/+^ littermate controls at E16.5

EDMA revealed statistically significant form differences in the mandible at E16.5, shown in Figure 4. Bootstrapped confidence intervals for all paired distances are given in Table S1, Supporting Information with significantly longer and shorter than expected distances identified. All linear distance measurements from the posterior-most landmark to each anterior landmark were shorter in *Fgfr2*^+/*P253R*^ mice compared to their *unaffected Fgfr2*^+/+^ *littermate controls*. Two linear distances at the anterior end of the mandible and linear distances between all three paired landmarks were larger in *Fgfr2*^+/*P253R*^ mice relative to *unaffected Fgfr2*^+/+^ *littermate controls*. Overall, mandibles of *Fgfr2*^+/*P253R*^ mice were smaller and wider than the mandibles of their *unaffected Fgfr2*^+/+^ *littermate controls*.

#### 3.2.2 | Morphological differences of *Fgfr2c*^*C342Y*/+^ Crouzon syndrome mice and unaffected *Fgfr2c*^+/+^ littermate controls at E16.5

At E16.5, mandibles of the *Fgfr2c*^*C342Y*/+^ Crouzon mice were significantly larger than those of their unaffected *Fgfr2c*^+/+^ littermate controls (Figure 5). Bootstrapped confidence intervals for all paired distances are given in Table S2. The majority of the linear distances taken on the left hemimandible and between the bilaterally paired landmarks were larger in *Fgfr2c*^*C342Y*/+^ mice compared to their *unaffected Fgfr2c*^+/+^ *littermate controls*.

### 3.3 | Mechanical strain and deformation on Meckel’s cartilage

The FEA results quantified deformation in the meshed models for each genotype as MC morphology changes from E14.5 to E16.5 using landmark, growth center, and combined approaches. Deformation is illustrated by two measurements: displacement, shown as vectors indicating the direction and magnitude of node movement, and equivalent strain (EQ), mapped onto the mesh to display the distribution of strain within MC. The results for each simulation method are described below.

#### 3.3.1 | FEA: Landmarks drive deformation in *Fgfr2*^+/*P253R*^ Apert syndrome mice

When landmarks are used to guide shape changes in *Fgfr2*^+/*P253R*^ Apert syndrome mice and their unaffected *Fgfr2*^+/+^ littermate controls, displacement was highest at the anterior and posterior ends of MC in both genotypes, and the magnitude of these displacements was higher in the *Fgfr2*^+/*P253R*^ specimen (Figure 6). The direction of the displacement vectors in the *Fgfr2*^+/+^ specimen had greater symmetry with respect to the sagittal plane when compared to those in the *Fgfr2*^+/*P253R*^ specimen, where displacement vectors had a greater left lateral component. EQ was highest at the anterior and posterior ends in both affected and unaffected specimens, and absolutely larger in the *Fgfr2*^+/*P253R*^ specimen with an asymmetrical distribution with respect to the sagittal plane.

#### 3.3.2 | FEA: Landmarks drive deformation in *Fgfr2c*^*C342Y*/+^ Crouzon syndrome mice

When landmarks are used to guide shape changes in the *Fgfr2c*^*C342Y*/+^ Crouzon syndrome mice and their unaffected *Fgfr2c*^+/+^ littermate controls, displacement is highest at the anterior end of the MC in the affected specimen, and highest in the arms of MC just behind the symphysis in the unaffected *Fgfr2c*^+/+^ specimen (Figure 7). The magnitude of the displacement vectors was larger in all areas of MC in the affected *Fgfr2c*^*C342Y*/+^ specimen compared to the unaffected *Fgfr2c*^+/+^ specimen. EQ was highest in the anterior and posterior ends in both *Fgfr2c*^*C342Y*/+^ and *Fgfr2c*^+/+^ specimens.

#### 3.3.3 | FEA: Growth centers drive deformation in the *Fgfr2*^+/*P253R*^ mouse model of Apert syndrome

When growth centers were used to guide shape changes between E14.5 and E16.5 to compare *Fgfr2*^+/*P253R*^ and *Fgfr2*^+/+^ mice, the greatest displacement was observed at the posterior ends of MC in both genotypes, though it was not as concentrated at the most posterior point as seen in the landmark-driven models (Figure 8). Displacement was greater in the *Fgfr2*^+/*P253R*^ specimen compared to the *Fgfr2*^+/+^ specimen. The direction of displacement vectors in the model for *Fgfr2*^+/+^ specimens exhibited greater symmetry relative to the sagittal plane when compared with the model for *Fgfr2*^+/*P253R*^ specimens. EQ was concentrated in and around the growth centers in both models, with the highest values observed in the *Fgfr2*^+/*P253R*^ model.

#### 3.3.4 | FEA: Growth centers drive deformation in the *Fgfr2c*^*C342Y*/+^ mouse model of Crouzon syndrome

When growth centers were used to characterize shape changes between E14.5 and E16.5 in the *Fgfr2c*^*C342Y*/+^ mouse model of Crouzon syndrome, the greatest displacement magnitudes occurred in the arms of MC in both genotypes, with displacement approximately twice as large in the *Fgfr2c*^*C342Y*/+^ specimen as the *Fgfr2c*^+/+^ specimen (Figure 9). Displacement vectors were generally symmetrical about the sagittal plane in both *Fgfr2c*^*C342Y*/+^ and *Fgfr2c*^+/+^ specimens. In the *Fgfr2c*^*C342Y*/+^ specimen, peak displacement extended throughout the arms of MC posterior to the growth centers, whereas in the *Fgfr2c*^+/+^ specimen, it was restricted to the posterior ends of the arms. EQ peaked in the arms near the growth centers in both *Fgfr2c*^*C342Y*/+^ and *Fgfr2c*^+/+^ specimens, but the magnitude of EQ was again approximately twice as large in the *Fgfr2c*^*C342Y*/+^ specimen.

#### 3.3.5 | FEA: Combination of landmark- and growth center-driven deformation

When landmark- and growth center-driven approaches are used together to guide shape change in the FEA models of the *Fgfr2*^+/*P253R*^ and *Fgfr2*^+/+^ specimens, the magnitude of displacement was highest in the anterior region of MC in the FEA models of both genotypes, with the *Fgfr2*^+/*P253R*^ FEA model indicating a marginally higher magnitude of displacement (Figure 10). The direction of displacement vectors is again starkly asymmetric about the sagittal plane in the FEA model of the *Fgfr2*^+/*P253R*^ specimen, but markedly less so in the FEA model of the *Fgfr2*^+/+^ specimen. The FEA model of the *Fgfr2*^+/*P253R*^ specimen has greater equivalent strain compared to the FEA model of the *Fgfr2*^+/+^ specimen. Equivalent strain is distributed more broadly throughout the anterior region of MC (anterior to growth centers) in the FEA models of both genotypes of the mouse model of Apert syndrome when the combined landmark- and growth center-driven models are compared to the landmark-driven and the growth center-driven models.

When the combined landmark- and growth center-driven approach was used to guide shape changes in the *Fgfr2c*^*C342Y*/+^ and *Fgfr2c*^+/+^ specimens, the magnitude of displacement was higher throughout MC in the FEA model of the *Fgfr2c*^+/+^ specimen (Figure 11). The highest displacements in both specimens were observed in the arms of MC, posterior to the growth centers, where the FEA model of the *Fgfr2c*^*C342Y*/+^ specimen exhibited nearly twice the displacement of the FEA model of the *Fgfr2c*^+/+^ specimen. EQ was highest in the arms of MC just posterior to the symphysis in the FEA models of both genotypes of the *Fgfr2c*^*C342Y*/+^ mouse model of Crouzon syndrome, whereas the magnitude of EQ was higher in the FEA model of the *Fgfr2c*^*C342Y*/+^ specimen.

Cross-sections were analyzed in the regions just posterior to the symphysis of MC, in the regions where EQ was highest, and views of EQ in MC from the FEA models that used the combined landmark- and growth center-driven approach, for FEA models of affected and unaffected genotypes of the mouse models of Apert and Crouzon syndromes (Figures 10 and 11). The cross-sectional views of EQ are paired with the histological sections used to identify hypertrophic cartilage in regions of MC that act as growth centers (Figure 12).

In the mouse model of Apert syndrome, EQ was greater and equally distributed throughout the cross-section for the *Fgfr2*^+/*P253R*^ specimen, whereas the highest values for EQ were found towards the center of the cross-section in the *Fgfr2*^+/+^ specimen. Histologically, the *Fgfr2*^+/+^ specimen had more centrally distributed hypertrophic chondrocytes compared to the *Fgfr2*^+/*P253R*^ specimen, where hypertrophy appeared more uniformly distributed throughout the cross-section.

In the mouse model of Crouzon syndrome, EQ was equivalent in the *Fgfr2c*^*C342Y*/+^ and *Fgfr2c*^+/+^ models, and generally evenly distributed throughout the cross-section. No major differences in the distribution of hypertrophic chondrocytes were evident between the histological sections from *Fgfr2c*^*C342Y*/+^ and *Fgfr2c*^+/+^ specimens of the mouse model of Crouzon syndrome in this region.

## 4 | DISCUSSION

This study characterizes the spatial distribution of strain and deformation within MC in FGFR2-associated models, demonstrating that biomechanical effects are regionally localized and substantively different between genotypes. The findings support the interpretation that MC is a structurally and functionally heterogeneous element of the embryonic mandible that may influence craniofacial morphogenesis through localized mechanobiological interactions rather than typical growth behavior (Hall, 1982a; Rodriguez-Vazquez et al., 1997). The mandible is a developmentally integrated composite structure in which MC provides early structural support and maintains a close spatial and functional relationship with the adjacent intramembranous bone (Motch Perrine et al., 2019; Pitirri et al., 2022). Consequently, region-specific alterations in MC mechanics may reflect or contribute to changes in mandibular ossification and morphology, including the mandibular dysmorphogenesis observed in FGFR2-related craniosynostosis (Motch Perrine et al., 2019; Pitirri et al., 2022).

### 4.1 | Regionalization of strain and its anatomical correlates

Strain was not uniformly distributed along MC in any model considered in this analysis but instead concentrated within either the growth center or the anterior region, or both, depending on the deformation approach that was used. The anterior region consistently exhibited the highest equivalent strain and deformation, whereas posterior regions, including the corpus and malleus, showed comparatively minimal mechanical response. This pattern was conserved across genotypes, indicating that regionalization of biomechanical activity is an intrinsic feature of MC during this developmental window (Kaucka et al., 2017; Shibata & Yokohama-Tamaki, 2008).

Anatomically, the growth center corresponds to a zone of active chondrocyte proliferation and hypertrophy, while the anterior region is active in cell proliferation (Foster et al., in review). The localization of strain to these regions suggests that they may function as biomechanical hotspots associated with growth-related processes that are particularly sensitive to mechanical perturbation. This interpretation is consistent with prior work demonstrating that cartilage growth is modulated by the local mechanical environment, even at relatively low strain magnitudes (Carter & Wong, 2003; Nowlan et al., 2010).

### 4.2 | Genotype-specific differences in strain magnitude and distribution

Although the spatial pattern of strain was broadly similar between affected and unaffected specimens for both Apert and Crouzon syndrome models, differences in magnitude and direction were evident. In the model for Crouzon syndrome, strain was localized to the growth center and anterior extremity region under the combined deformation approach, with peak values of approximately 0.032 mm/mm. In contrast, the Apert model exhibited higher peak strain, approaching 0.04 mm/mm, and a more extensive distribution across the anterior region, particularly within and surrounding the growth center area (GCA).

These findings suggest that FGFR2 sequence variants are associated with differential effects on the mechanical environment of MC. The broader and more elevated strain distribution observed in the Apert syndrome model, particularly in the affected specimen, suggests that altered MC morphology and localized growth activity interact to shape the mechanical environment of the developing mandibular precursor. This interpretation is consistent with biomechanical models of cranial growth showing that dimensional, geometric, and physical constraints can influence regional tissue deformation during skull development (Weickenmeier et al., 2017). Conversely, the more localized strain pattern in the Crouzon syndrome model may reflect comparatively constrained perturbation of MC biomechanics. Together, the Apert and Crouzon syndrome results suggest that growth centers may act as critical loci for mechanotransduction, where localized changes in cellular activity, cartilage geometry, and adjacent skeletal constraints are translated into divergent strain patterns during development. This interpretation is consistent with evidence from craniosynostosis models showing that altered cranial joint loading can modify craniofacial phenotype severity, supporting the broader principle that local mechanical environments contribute to genotype-specific craniofacial outcomes (Moazen et al., 2022), and aligns with evidence that distinct FGFR2 variants produce differential effects on craniofacial development, potentially through differences in signaling intensity, spatial expression, or downstream cellular responses (Eswarakumar et al., 2004; Holmes & Basilico, 2012).

### 4.3 | Biological relevance of low-magnitude strain

The absolute strain magnitudes observed in this study are modest (<0.04) yet may be biologically meaningful. Developing cartilage is highly mechanosensitive and small changes in mechanical loading can influence chondrocyte proliferation, differentiation, and extracellular matrix organization (Carter & Wong, 2003; Nowlan et al., 2010). Importantly, the spatial concentration of strain within anatomically critical regions may be more consequential than magnitude alone.

Localized increases in strain within the growth center and anterior region may represent a mechanism by which subtle early perturbations could influence mandibular morphogenesis. Over developmental time, repeated or sustained mechanical effects in these regions could potentially alter growth trajectories, contributing to differences in mandibular size, symmetry, and shape. This interpretation is consistent with mechanobiological models of skeletal development in which local mechanical cues guide tissue differentiation and morphogenesis (Hall, 1982a; Nowlan et al., 2010).

### 4.4 | Regional specialization of Meckel’s cartilage

The consistent observation of low strain and deformation in posterior MC regions across all models and deformation approaches supports the concept of regional specialization along the length of the cartilage. The corpus and otic regions are associated with distinct developmental outcomes, including contribution to the mandibular body and middle ear ossicles, respectively (Rodriguez-Vazquez et al., 1997). Their relative mechanical stability may be related to differences in material properties, cellular organization, or timing of developmental activity. Alternatively, these regions may be less directly influenced by early mandibular growth dynamics or mechanical loading conditions. The present findings therefore reinforce the view that MC is not a homogeneous structure but comprised of anatomically and functionally distinct sub-regions with differential sensitivity to biomechanical forces (Kaucka et al., 2017).

## 5 | INTEGRATION OF BIOMECHANICS AND MANDIBULAR DEVELOPMENT

The alignment of strain and deformation patterns with known growth regions suggests a possible integration between the mechanical environment and developmental processes within MC. Increased strain in the growth center may influence chondrocyte hypertrophy and matrix deposition while deformation in the anterior region may affect midline integration. The mechanical behavior of cartilage has been shown to vary by composition and in association with adjacent bone, and a link between mechanical stress on cartilage and bone growth is well known (Moazen et al., 2022; Weickenmeier et al., 2017). Given this, the localized effects described here for MC can provide a mechanistic framework linking altered cartilage mechanics to mandibular dysmorphogenesis.

Notably, this interpretation does not require persistent structural differences in MC. Instead, transient alterations in growth dynamics and the mechanical environment may be associated with changes in downstream bone formation. Such a model is consistent with observations that early developmental perturbations can have lasting effects on craniofacial morphology even when initial structural differences are no longer apparent at later stages (Hall, 1982b; Shibata & Yokohama-Tamaki, 2008).

### 5.1 | Localized variability

Localized regions of low strain observed on the unaffected specimens, particularly in the FEA model of the mouse model of Apert syndrome, suggest the presence of subtle asymmetries in the mechanical environment or tissue properties. While not directly quantified here, asymmetries like these may contribute to variation in mandibular morphology and could represent an additional mechanism by which genotype influences phenotype. Further investigations using higher resolution and longitudinal approaches with larger sample sizes are required to clarify these effects.

### 5.2 | Limitations

Several limitations should be considered when interpreting these findings. Our sample sizes were limited, which limited our ability to perform additional cross model statistical analyses. The finite element models use simplified and homogeneous material properties, which may not fully represent the complex biomechanics of embryonic cartilage. Strain and deformation were inferred from modeled displacement rather than measured directly in vivo, and the relationship between mechanical strain and cellular processes such as proliferation, differentiation, and matrix remodeling was not directly assessed.

## 6 | CONCLUSIONS AND FUTURE DIRECTIONS

Biomechanical strain within MC appears to be regionally localized and concentrated in anatomically significant growth regions. Differences in strain magnitude and distribution between FGFR2 models for Crouzon and Apert syndromes suggest that substituted sequence-specific effects on cartilage mechanics may be associated with variation in mandibular phenotypes. These findings underscore the importance of regional anatomy and mechanical environment in craniofacial development.

Future work integrating biomechanical modeling with histological and molecular analyses will be essential to define how localized strain influences cellular behavior within MC. Incorporation of heterogeneous material properties and more physiologically realistic loading conditions will further refine these models. Extending these analyses across developmental stages may provide additional insight into how early biomechanical differences translate into mature craniofacial forms.

## Supplementary Material

Supplementary Materials

Additional supporting information can be found online in the Supporting Information section at the end of this article.

## Figures and Tables

**FIGURE 1:**
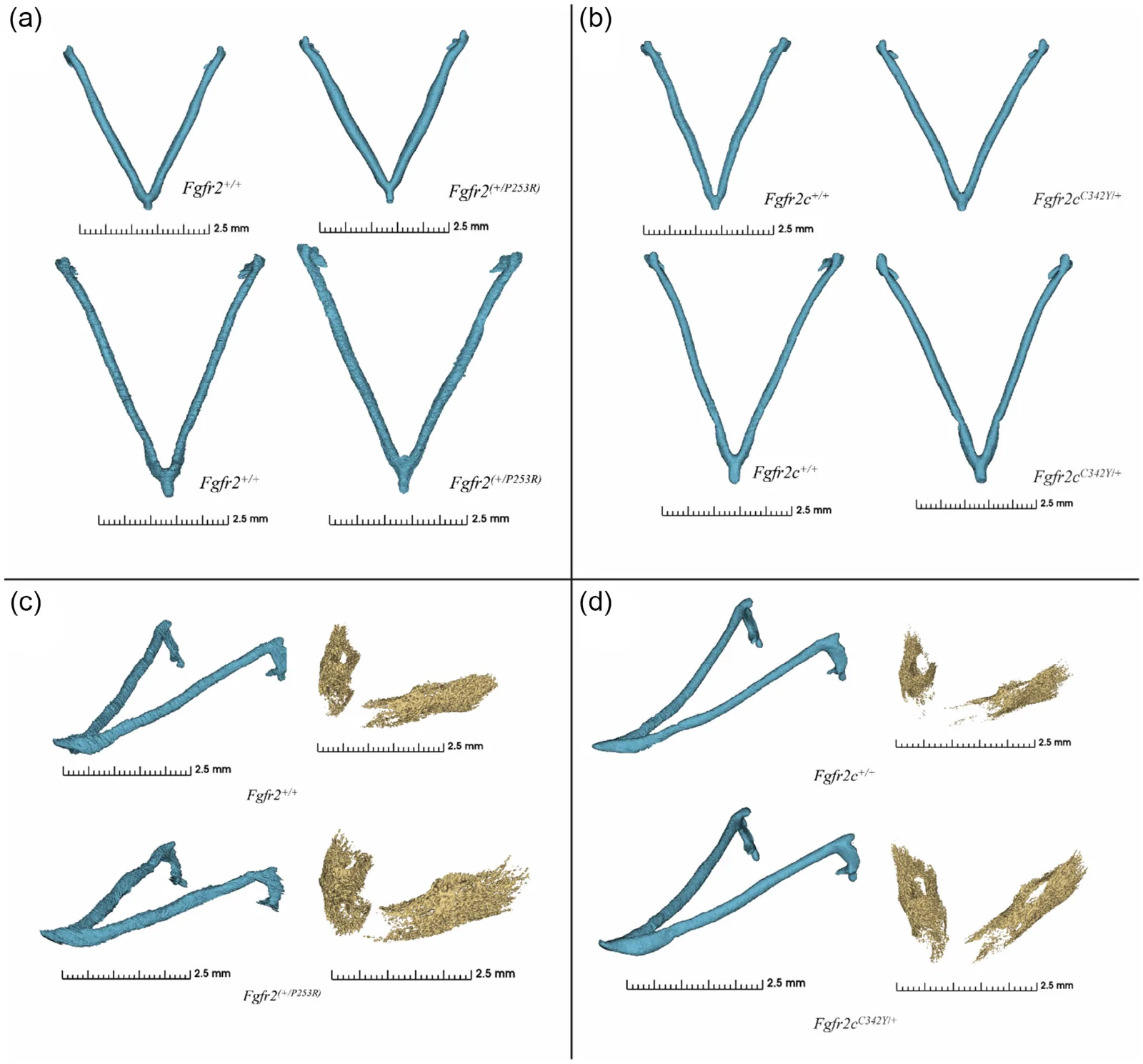
Representative Meckel’s cartilage and mandibular morphology in specimens of the mouse models of Apert and Crouzon syndromes. (a) Dorsal/superior views of 3D renderings of Meckel’s cartilage (MC) from unaffected (*Fgfr2*^+/+^) and affected Apert model (*Fgfr2*^+/*P253R*^) specimens at E14.5 (top row) and E16.5 (bottom row). (b) Dorsal/superior views of MC from unaffected (*Fgfr2c*^+/+^) and affected Crouzon model (*Fgfr2c*^*C342Y*/+^) specimens at E14.5 (top row) and E16.5 (bottom row). (c, d) Three-quarter frontal views of MC (blue) and mandibular bone (tan) at E16.5 in unaffected (top row) and affected (bottom row) Apert and Crouzon model specimens, respectively. Scale bar = 2.5 mm.

**FIGURE 2:**
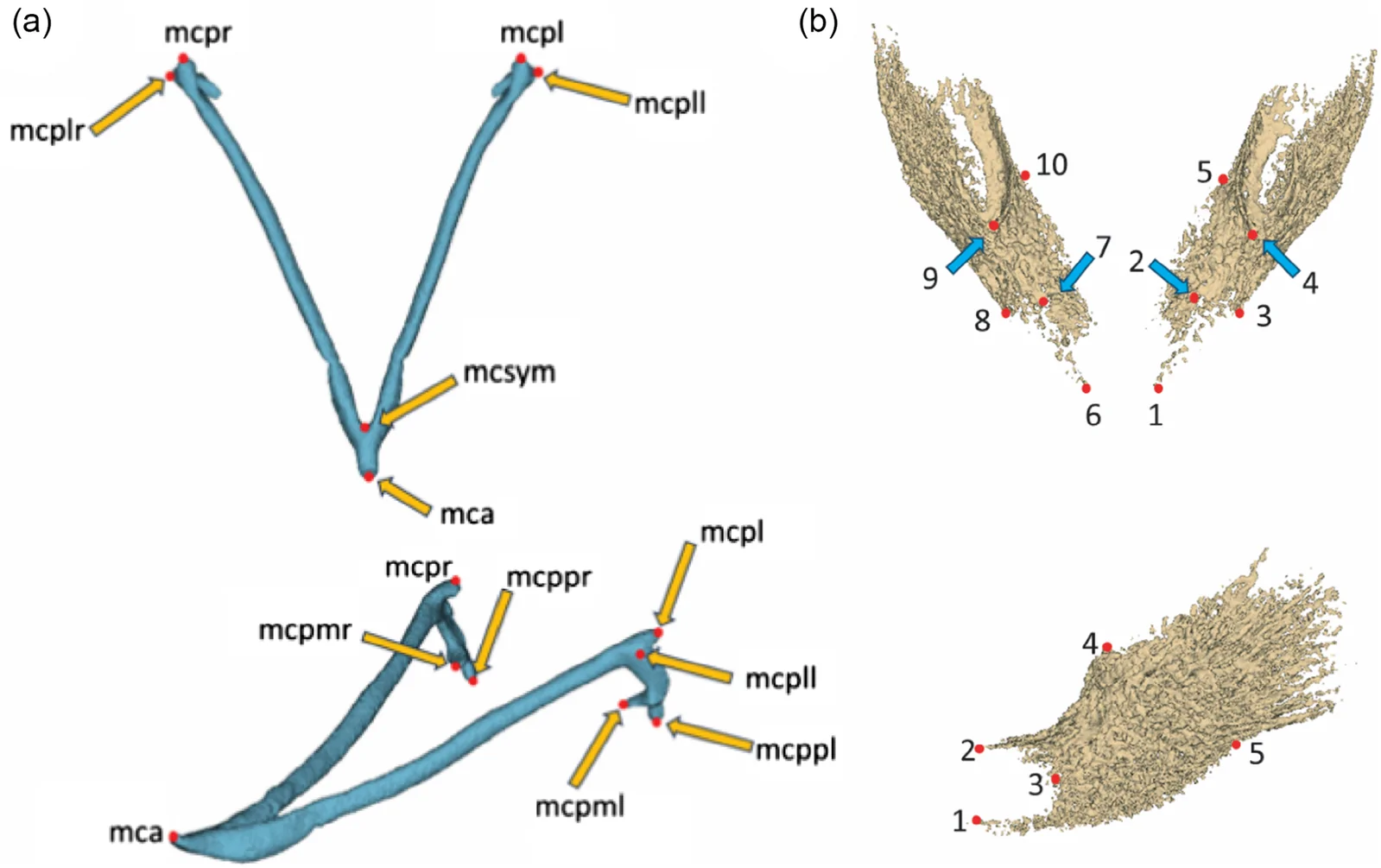
Anatomical landmarks used for Meckel’s cartilage and mandibular EDMA. Anatomical landmarks placed on 3D renderings of Meckel’s cartilage (MC; blue) and mandibular bone (tan). MC and mandibular landmarks are defined in Tables 3 and 4.

**FIGURE 3:**
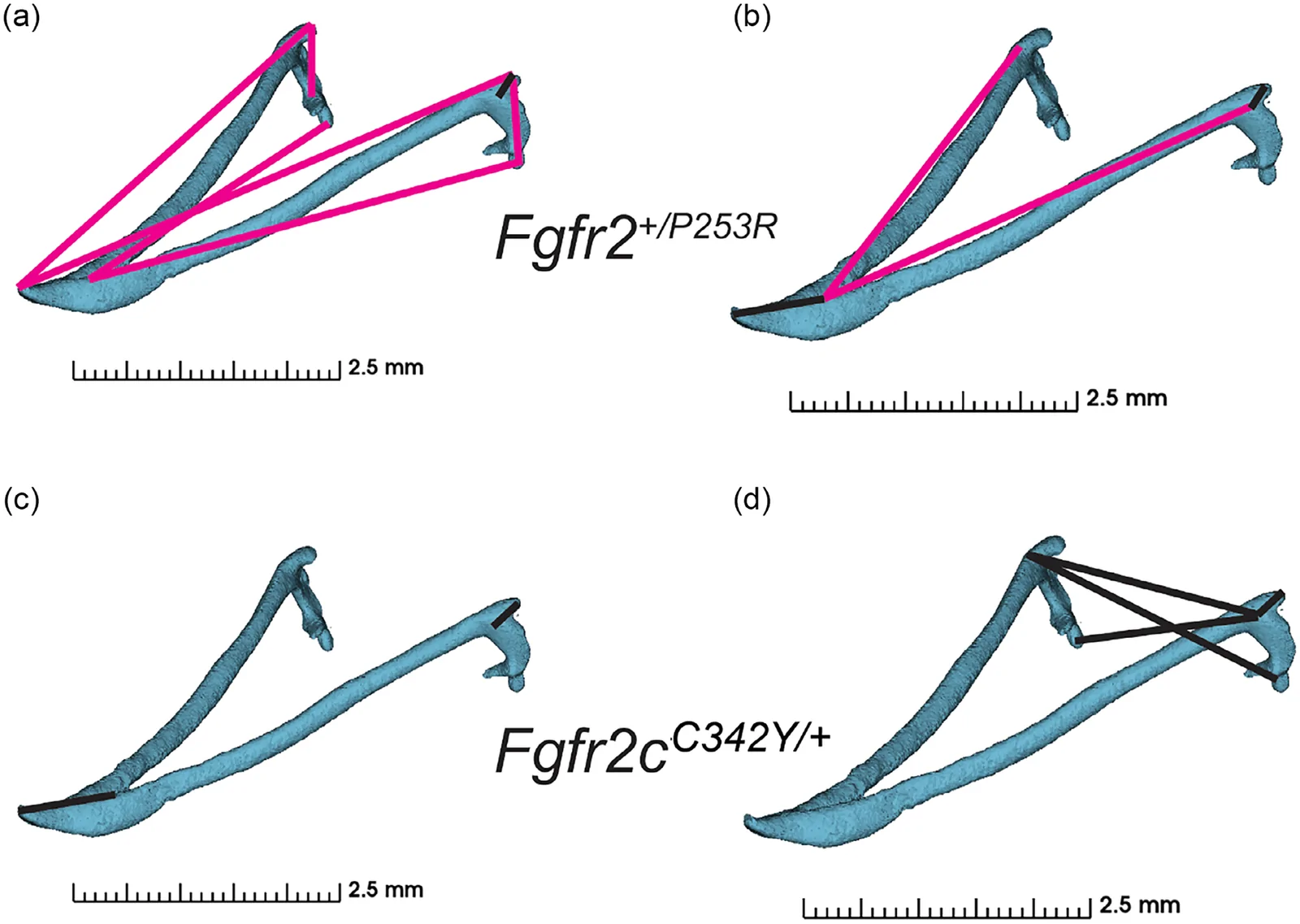
EDMA results for Meckel’s cartilage in mouse models of Apert and Crouzon syndromes. Results of Euclidean Distance Matrix Analysis (EDMA) comparing affected and unaffected specimens. Apert model differences are shown at E14.5 (a) and E16.5 (b); Crouzon model differences are shown at E14.5 (c) and E16.5 (d). Pink lines indicate linear distances that are significantly shorter in affected specimens compared as compared to their appropriate unaffected littermate controls. Black lines indicate linear distances that are significantly longer in affected specimens compared as compared to their appropriate unaffected littermate controls. Scale bars = 2.5 mm.

**FIGURE 4:**
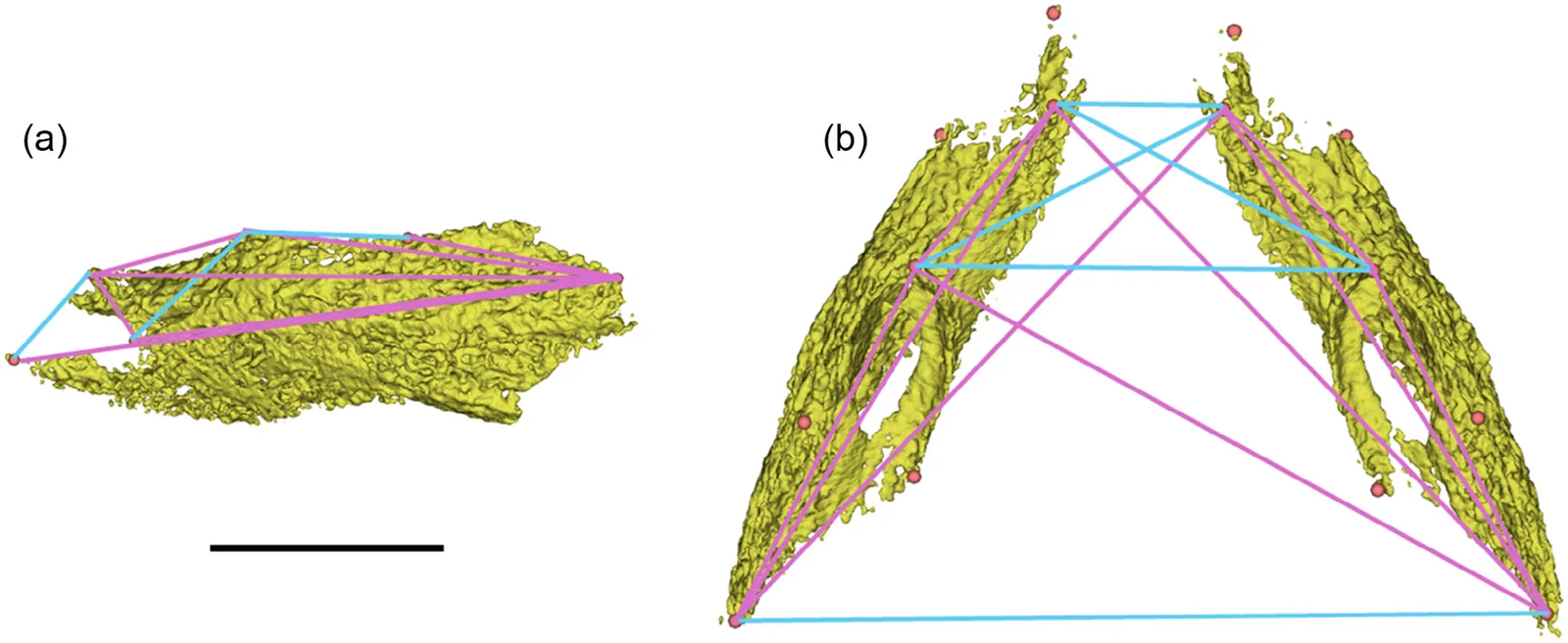
Mandibular form differences in the mouse model of Apert syndrome at E16.5. EDMA results comparing mandibular linear distances between affected Apert model *Fgfr2*^+/*P253R*^ and *Fgfr2*^+/+^ specimens at E16.5. A representative unaffected mandible is shown in left lateral view (a) and superior view (b). Pink lines indicate linear distances that are significantly shorter in affected specimens compared as compared to their appropriate unaffected littermate controls. Blue lines indicate linear distances that are significantly longer in affected specimens compared as compared to their appropriate unaffected littermate controls. Scale bar = 1 mm.

**FIGURE 5:**
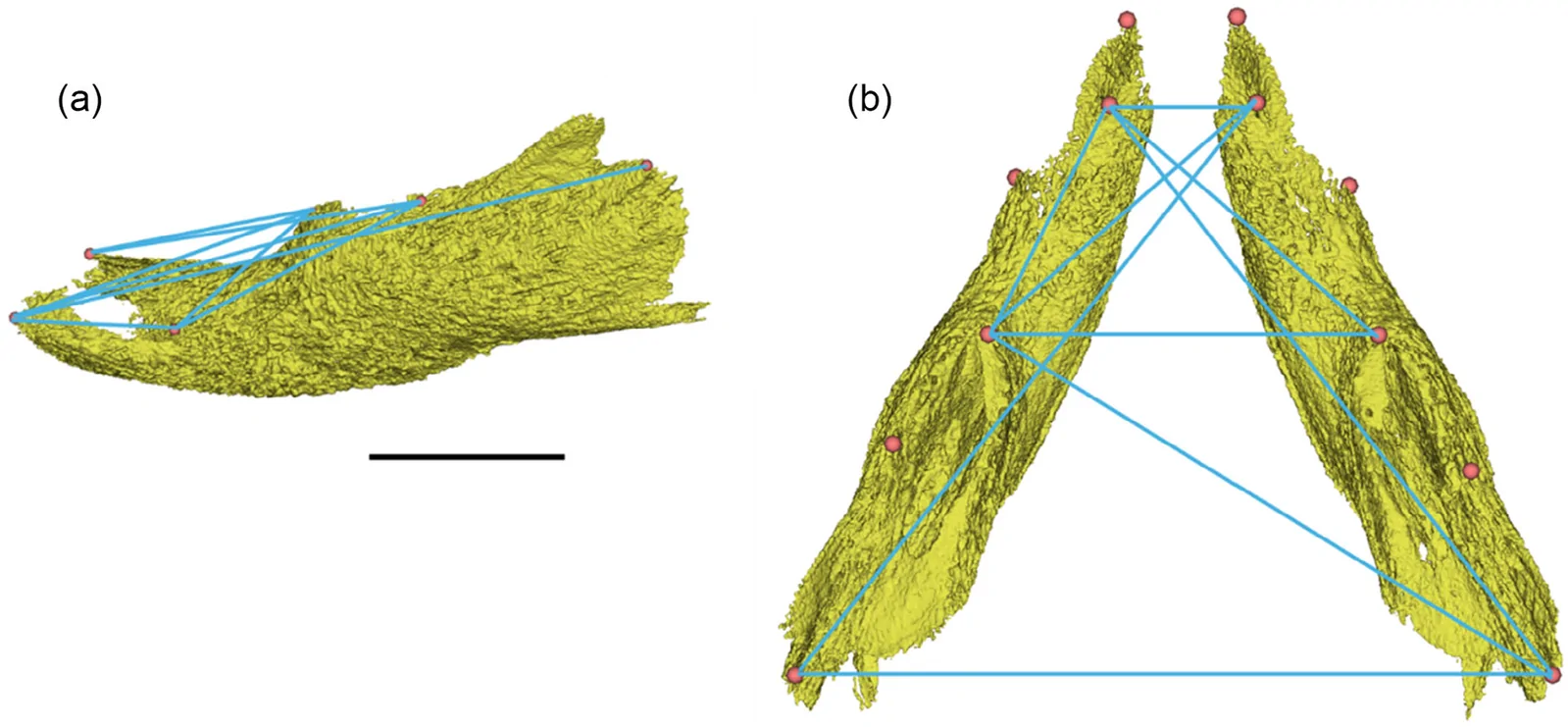
Mandibular form differences in the mouse model of Crouzon syndrome at E16.5. Results of EDMA analysis comparing linear distances between landmarks on Crouzon model *Fgfr2c*^*C342Y*/+^ and *Fgfr2c*^+/+^ specimens at E16.5. A 3D model of a representative unaffected specimen is shown in left lateral (a) and superior (b) view. Linear distances that are shorter in affected compared to unaffected specimens are shown in pink. Linear distances that are longer in affected compared to unaffected specimens are shown in blue. Black scale bar = 1 mm.

**FIGURE 6:**
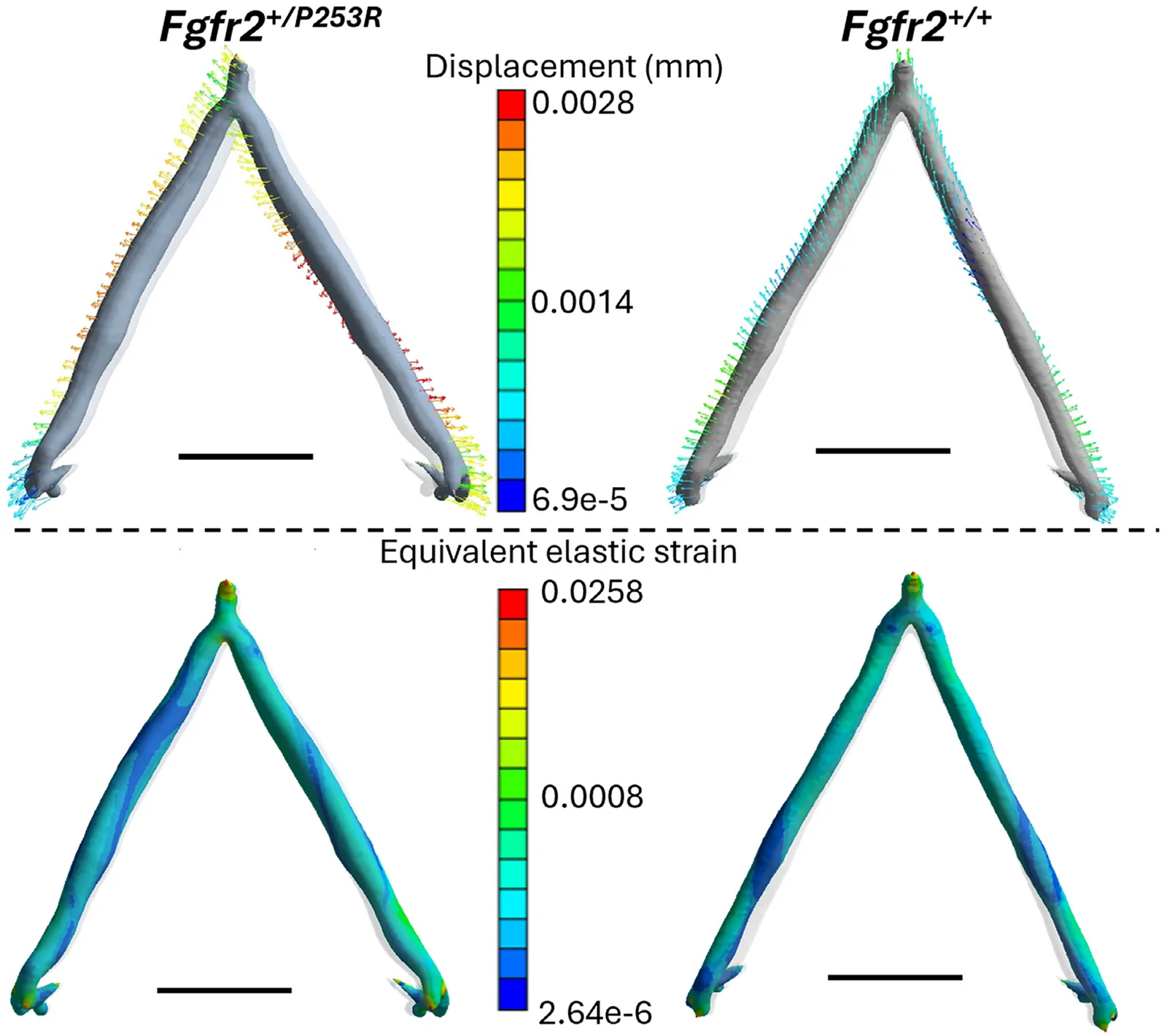
Deformation results from landmark displacement method on MC in mouse model of Apert syndrome *Fgfr2*^+/*P253R*^ and *Fgfr2*^+/+^ specimens. Top row shows displacement vectors plotted on meshed models for affected (top left) and unaffected (top right) where arrows show the direction of deformation and color shows the magnitude corresponding to the legend (top center). Bottom row shows color maps that illustrate the distribution of equivalent elastic strain across the surface of meshed models for affected (bottom left) and unaffected (bottom right) where color shows the magnitude corresponding to the legend (bottom center). Black scale bar = 1 mm.

**FIGURE 7:**
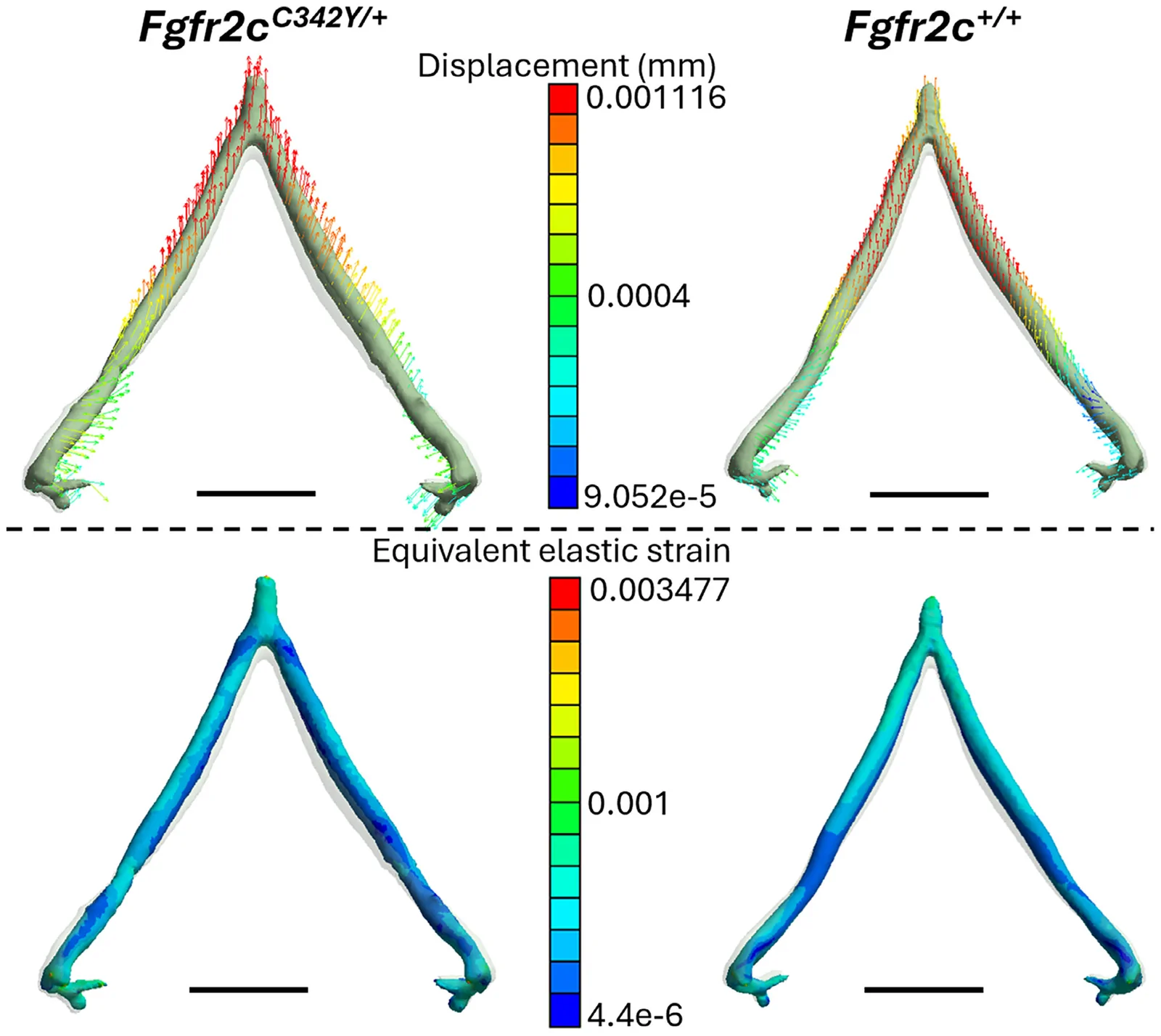
Deformation results from landmark displacement method on MC in mouse model of Crouzon syndrome *Fgfr2c*^*C342Y*/+^ and *Fgfr2c*^+/+^ specimens. Top row shows displacement vectors plotted on meshed models for affected (top left) and unaffected (top right) where arrows show the direction of deformation and color shows the magnitude corresponding to the legend (top center). Bottom row shows color maps that illustrate the distribution of equivalent elastic strain across the surface of meshed models for affected (bottom left) and unaffected (bottom right) where color shows the magnitude corresponding to the legend (bottom center). Black scale bar = 1 mm.

**FIGURE 8:**
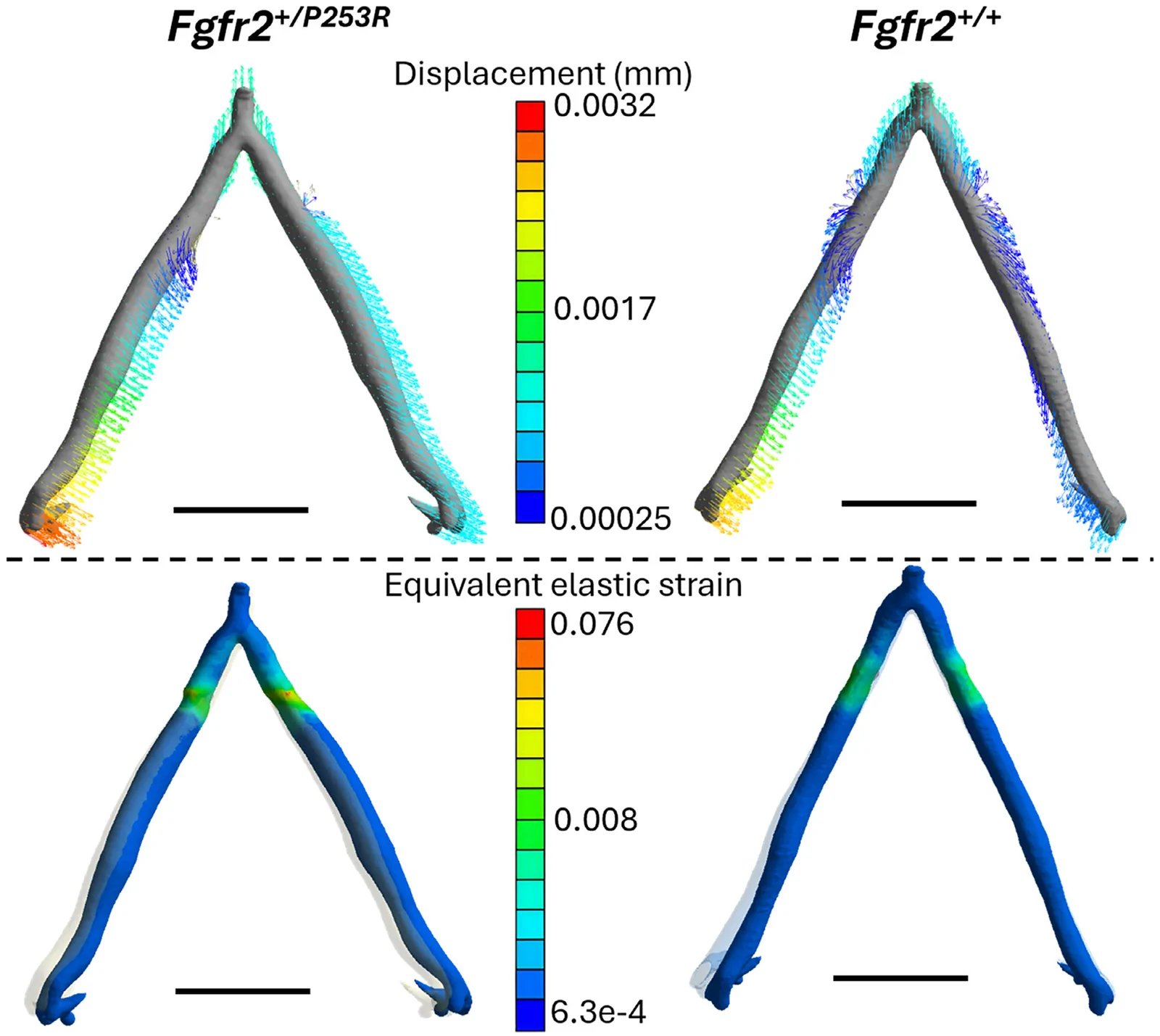
Deformation results from growth center displacement method on MC in mouse model of Apert syndrome *Fgfr2*^+/*P253R*^ and *Fgfr2*^+/+^ specimens. Top row shows displacement vectors plotted on meshed models for affected (top left) and unaffected (top right) where arrows show the direction of deformation and color shows the magnitude corresponding to the legend (top center). Bottom row shows color maps that illustrate the distribution of equivalent elastic strain across the surface of meshed models for affected (bottom left) and unaffected (bottom right) where color shows the magnitude corresponding to the legend (bottom center). Black scale bar = 1 mm.

**FIGURE 9:**
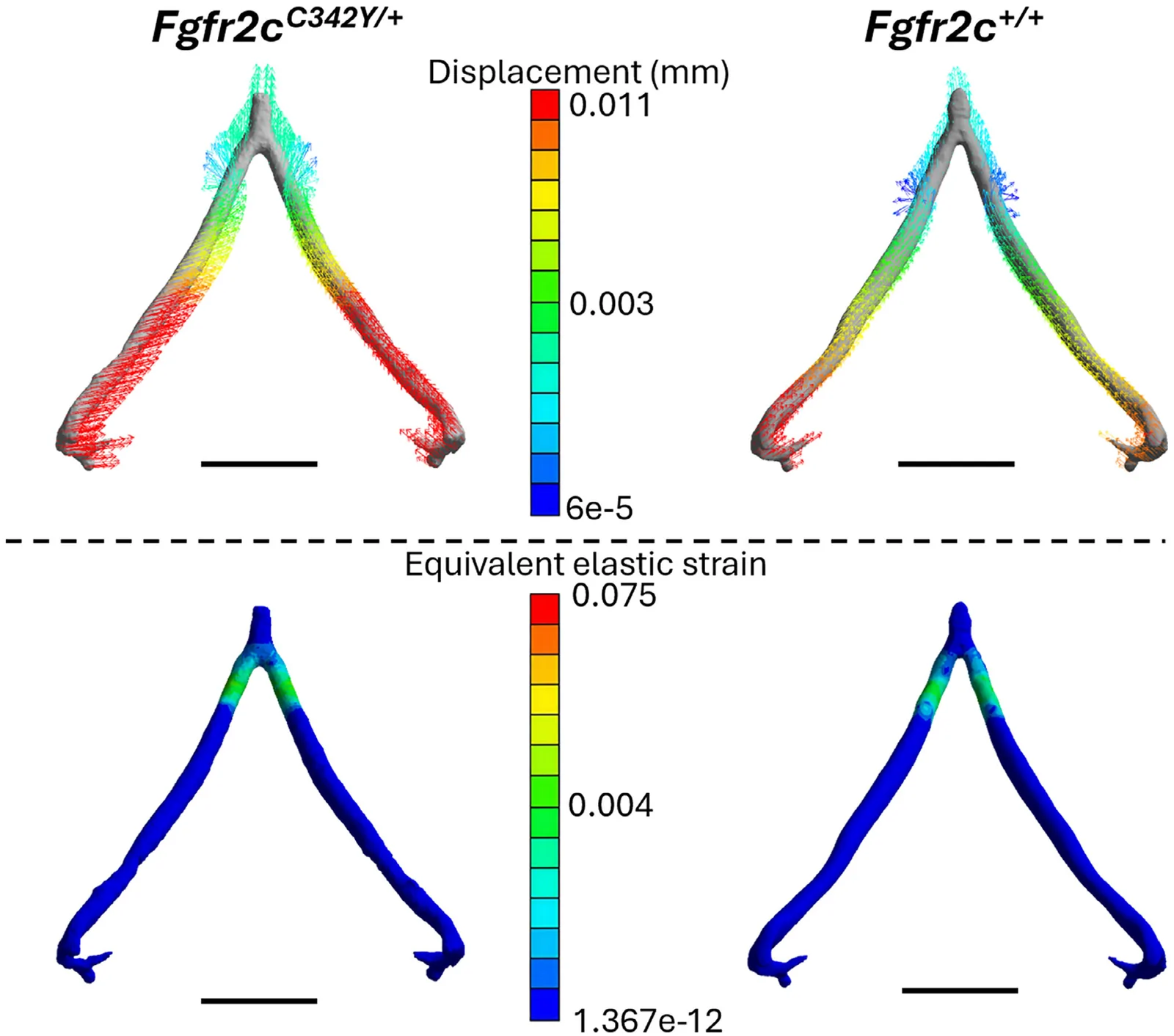
Deformation results from growth center displacement method on MC in mouse model of Crouzon syndrome *Fgfr2c*^*C342Y*/+^ and *Fgfr2c*^+/+^ specimens. Top row shows displacement vectors plotted on meshed models for affected (top left) and unaffected (top right) where arrows show the direction of deformation and color shows the magnitude corresponding to the legend (top center). Bottom row shows color maps that illustrate the distribution of equivalent elastic strain across the surface of meshed models for affected (bottom left) and unaffected (bottom right) where color shows the magnitude corresponding to the legend (bottom center). Black scale bar = 1 mm.

**FIGURE 10:**
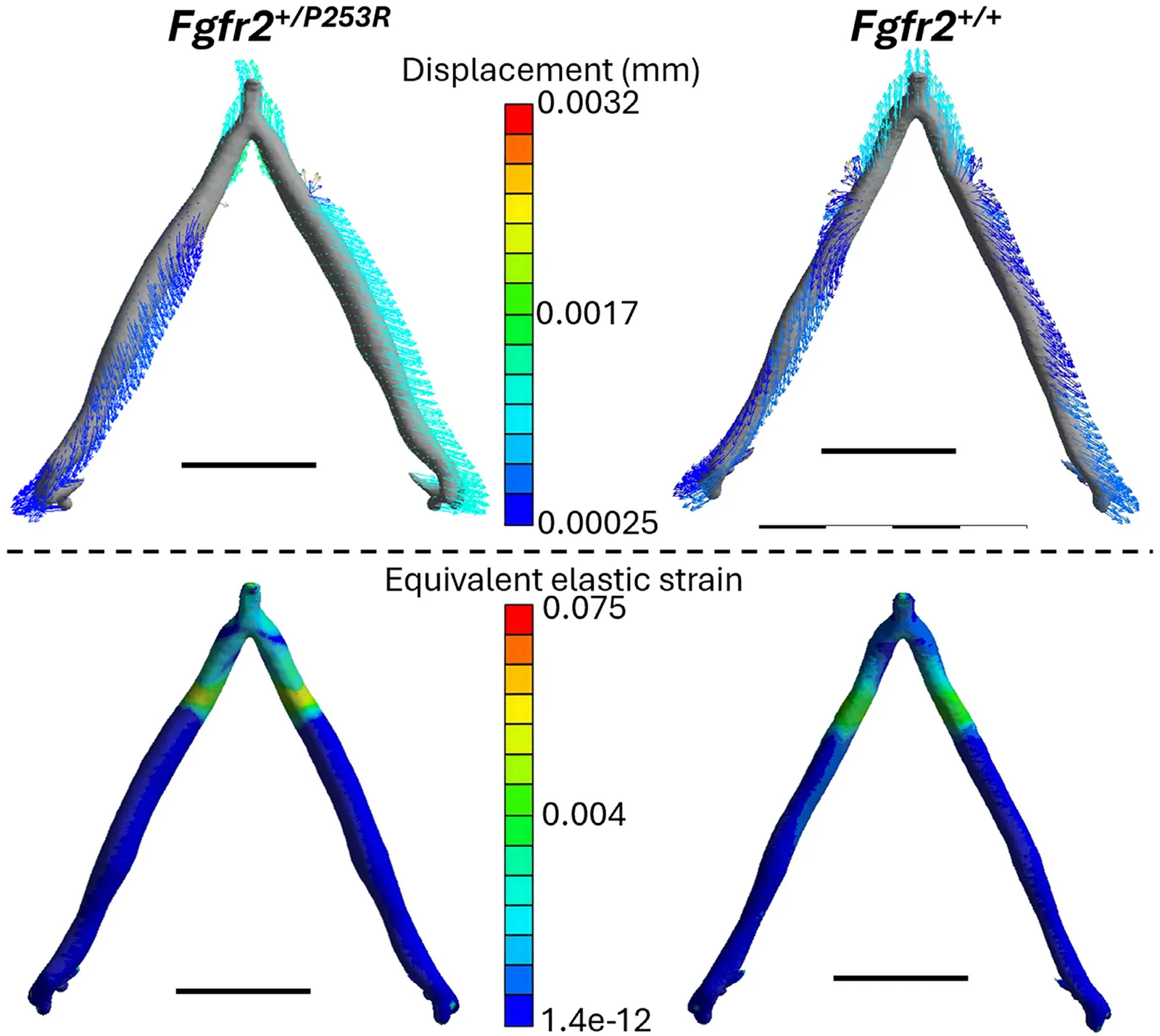
Deformation results from combined landmark displacement and method and growth center displacement method on MC in mouse model of Apert syndrome *Fgfr2*^+/*P253R*^ and *Fgfr2*^+/+^ specimens. Top row shows displacement vectors plotted on meshed models for affected (top left) and unaffected (top right) where arrows show the direction of deformation and color shows the magnitude corresponding to the legend (top center). Bottom row shows color maps that illustrate the distribution of equivalent elastic strain across the surface of meshed models for affected (bottom left) and unaffected (bottom right) where color shows the magnitude corresponding to the legend (bottom center). Black scale bar = 1 mm.

**FIGURE 11:**
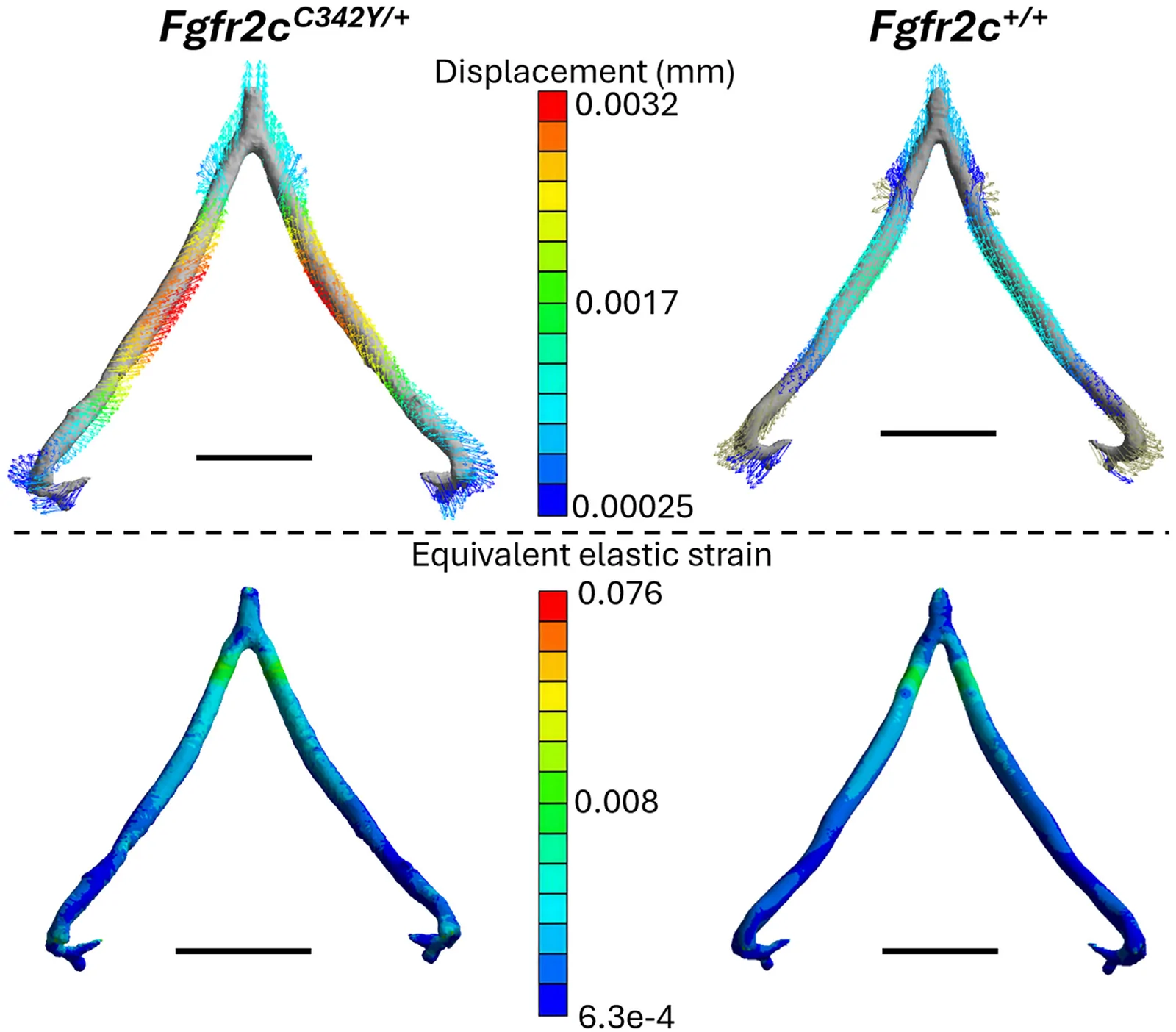
Deformation results from combined landmark displacement and method and growth center displacement method on MC in mouse model of Crouzon syndrome *Fgfr2c*^*C342Y*/+^ and *Fgfr2c*^+/+^ specimens. Top row shows displacement vectors plotted on meshed models for affected (top left) and unaffected (top right) where arrows show the direction of deformation and color shows the magnitude corresponding to the legend (top center). Bottom row shows color maps that illustrate the distribution of equivalent elastic strain across the surface of meshed models for affected (bottom left) and unaffected (bottom right) where color shows the magnitude corresponding to the legend (bottom center). Black scale bar = 1 mm.

**FIGURE 12:**
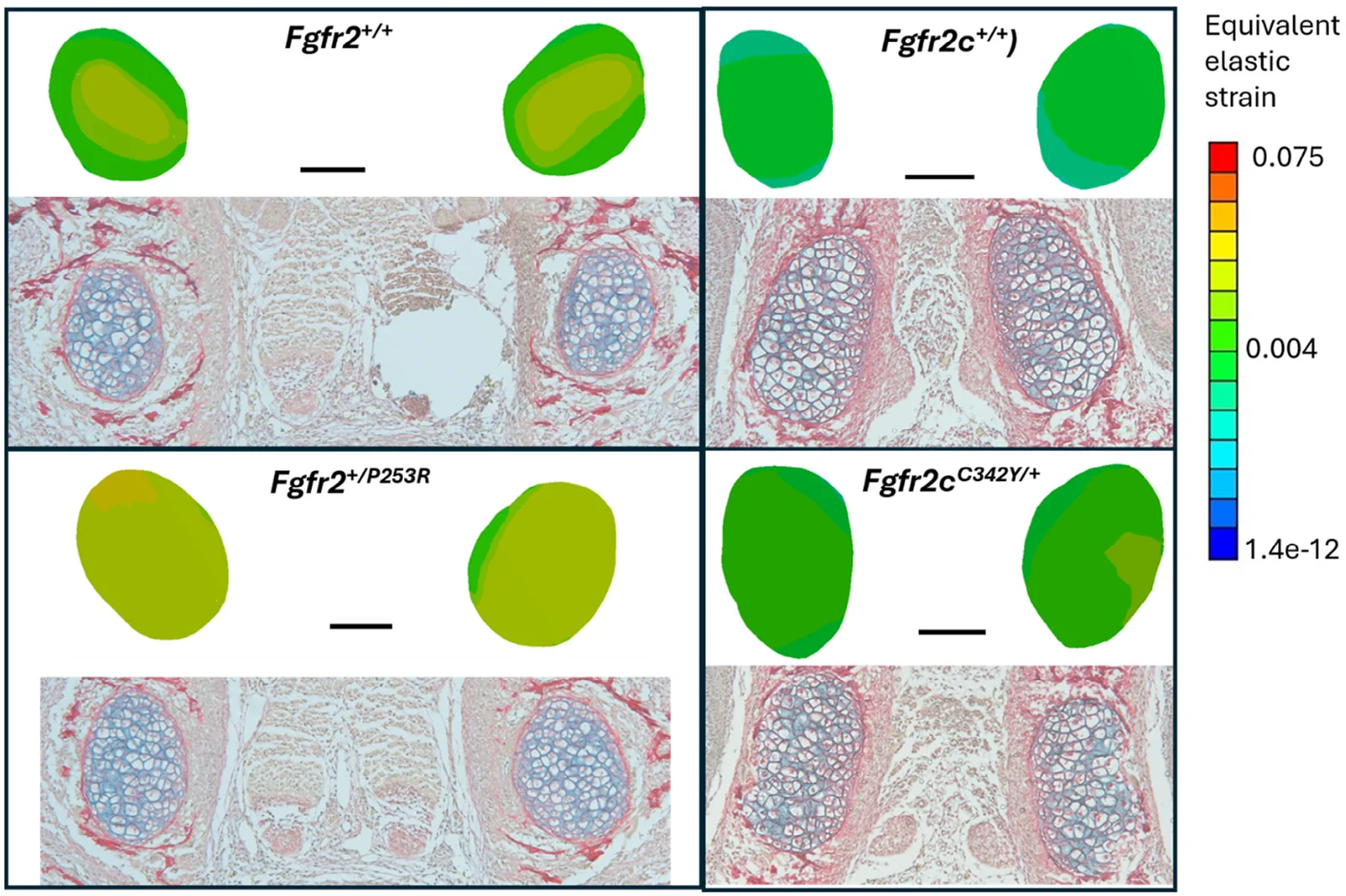
Comparison of FEA results for combined landmark and growth center deformation models with histological results showing chondrocyte cell size. A coronal cross-section is shown for each genotype through MC, posterior to the symphysis. The FEA results (green) show the distribution of EQ in MC and the histological sections show the chondrocytes in an approximately equivalent region of MC. Black scale bar = 1 mm.

**TABLE 1:** Sample size for each group.

Human condition	Genotype of mouse model	Age		
		E14.5	E16.5	Total
Apert syndrome	*Fgfr2^(+/P253R)^*	5	5	10
	*Fgfr2^(+/+)^*	4	5	9
Crouzon syndrome	*Fgfr2c* ^*(C342Y/+)*^	8	7	15
	*Fgfr2c^(+/+)^*	7	5	12
Total		24	22	46

**TABLE 2:** Micro-CT scan settings.

Mouse model	Specimen age	Bone scan parameters			PTA-e scan parameters		
		kV	μA	Average voxel size (μm)	kV	μA	Average voxel size (μm)
*Fgfr2* ^+/*P253R*^	E14.5			-	80	160	7.2
	E16.5	80	220	7.0	80	220	7.0
*Fgfr2c* ^*C342Y*/+^	E14.5			-	60	170–240	4.5
	E16.5	60–70	100	8.0	60	110–170	8.0

**TABLE 3:** Landmarks defining mandibular morphology.

Abbreviation	Description
1, m-lini	Inferior-most point on incisor alveolar rim at midline of the incisor at bone-tooth junction, left side
2, m-lsym	Junction of the rim of the alveolar process with left incisor, most lateral and centered along the cranial-caudal axis (taken on bone not tooth)
3, m-lsin	Superior-most point on incisor alveolar rim at midline of bone-tooth junction, left side
4, m-lamol	Anterior-lateral edge of alveolar process where first molar hits alveolus (anterior molar alveolus), left side
5, m-lacp	Intersection of molar alveolar rim and base of coronoid process (posterior molar alveolus), left side
6, m-lacond	Most caudal point on the cranial angle of the condyloid process, left side
7, m-rini	Inferior-most point on incisor alveolar rim at midline of the incisor at bone-tooth junction, right side
8, m-rsym	Junction of the rim of the alveolar process with right incisor, most lateral and centered along the cranial-caudal axis (taken on bone not tooth)
9, m-rsin	Superior-most point on incisor alveolar rim at midline of bone-tooth junction, right side
10, m-ramol	Anterior-lateral edge of alveolar process where first molar hits alveolus (anterior molar alveolus), right side
11, m-lrvmacp	Intersection of molar alveolar rim and base of coronoid process (posterior molar alveolus), right side
12, m-racond	Most caudal point on the cranial angle of the condyloid process, right side)

**TABLE 4:** Landmarks defining MC morphology.

Abbreviation	Description
mca	Most anterior point on the anterior extremity (tip) of MC
mcsym	Most posterosuperior point on the anterior extremity of MC
mcpl	Most posterior point on the left arm of MC
mcppl	Most inferior point on the posterior aspect of the left arm of MC
mcpll	Most lateral point on the posterior aspect of the left arm of MC
mcpml	Most medial point on the posterior aspect of the left arm of MC
mcpr	Most posterior point on the right arm of MC
mcppr	Most inferior point on the posterior aspect of the right arm of MC
mcplr	Most lateral point on the posterior aspect of the right arm of MC
mcpmr	Most medial point on the posterior aspect of the right arm of MC

## Data Availability

The data that support the findings of this study are available from the corresponding author upon reasonable request.
