# Supplementary material for: Biomechanical strain patterns in Meckel’s cartilage underlie mandibular variation in FGFR2 mouse models
Source: Anat Rec (Hoboken). Author manuscript; Available in PMC 2026 Oct 22. (PMC13688089; doi:10.1002/ar.70371)
Supplement: Supplementary file 1 [file NIHMS2216842-supplement-Supplementary_Materials.docx]

***Table S1: EDMA results comparing specimens of the mouse model of Apert syndrome affected (Fgfr2^+/P253R^) and genetically unaffected (Fgfr2^+/+^) mandibles at E16.5.*** *Linear distances between landmark pairs that are significantly different than expected are highlighted in pink (shorter) and blue (longer), and are associated with distances shown in Figure 4.*

***Table S2: EDMA results comparing specimens of the mouse model of Crouzon syndrome affected (Fgfr2c^C342Y/+^) and genetically unaffected (Fgfr2c^+/+^) mandibles at E16.5.*** *Linear distances between landmark pairs that are significantly different than expected are highlighted in blue (longer) and are associated with distances shown in Figure 5. Note: there are no significantly shorter distances than expected.*
